# Nonclinical Safety Evaluation of Nonmutagenic Immunogrit Following Subacute 28‐Day Repeated Dose Oral Toxicity in Sprague–Dawley Rats With GLP Compliance

**DOI:** 10.1155/jt/2786338

**Published:** 2026-07-18

**Authors:** Acharya Balkrishna, Kunal Bhattacharya, Sudeep Verma, Himanshu Jangid, Savita Lochab, Anurag Varshney

**Affiliations:** ^1^ Drug Discovery and Development Division, Patanjali Research Foundation, Haridwar 249 405, Uttarakhand, India, patanjaliresearchfoundation.com; ^2^ Department of Allied and Applied Sciences, University of Patanjali, Patanjali Yog Peeth, Haridwar 249 405, Uttarakhand, India, universityofpatanjali.com; ^3^ Patanjali Yog Peeth (UK) Trust, 40 Lambhill Street Kinning Park, Glasgow G41 1AU, UK

**Keywords:** ames test, ayurveda, immunogrit, sprague-dawley rats, subacute toxicity

## Abstract

Immunogrit is a traditional Ayurvedic medicine prescribed to patients for their general well‐being. In this study, Immunogrit was evaluated for its subacute (28 days) repeated oral dose toxicity in male and female Sprague‐Dawley (SD) rats of similar age and body weight following OECD 407 guidelines under Good Laboratory Practices compliance. Mutagenic potential of Immunogrit was also evaluated through Ames assay following OECD 471 guidelines. Phytochemical profiling of the Immunogrit was performed using ultraperformance liquid chromatography coupled with electrospray ionization quadrupole time‐of‐flight(UPLC/MS‐QToF) tandem mass spectrometer. UPLC/MS‐QToF analysis of Immunogrit identified 74 phytochemicals, well known for their health benefits. Male and female specific pathogen–free SD rats were orally gavaged to 100, 300, and 1000 mg/kg body weight/day of Immunogrit, over a period of 28 days, with two additional groups for a recovery phase of 14 days. No incidence of mortality, morbidity, or abnormal clinical signs was observed in the Immunogrit‐exposed animals. Body weight, behavioral, and food consumption habit were found to be normal throughout the study period. Immunogrit exposure did not induce ophthalmic, neurological, functional, hematological, biochemical, and histopathological irregularities in animals over a period of 28 days. Latent incidence of toxicity was also not observed in the recovery group male and female rats kept under observation for an additional 14 days’ post‐Immunogrit exposure. Ames test of Immunogrit and its S9‐treated fraction in *Salmonella typhimurium* strains TA1535, TA1537, TA98, and TA100, and *Escherichia coli* WP2uvrA did not show any induced mutagenic potential, up to the highest dose tested. In conclusion, Immunogrit was found to be nonmutagenic and showed a “no‐observed‐adverse‐effect‐level” (NOAEL) of 1000 mg/kg body weight/day in male and female SD rats.

## 1. Introduction

Herbal therapeutics are an integral part of the classical medicinal system applied worldwide throughout human history for their recorded health benefits. Therapeutic applications of herbal medicines have promoted immune resistance of the human body against external stimuli and environmental factors [1, 2]. According to a published World Health Organization (WHO) report, approximately 80% of the world’s population currently uses herbal remedies in traditional forms for treating various ailments [3]. This makes it critical to perform the toxicological evaluation of the herbal medicines to elude any immediate or latent side‐effects [4]. Several herbal supplements and remedies have been reported to induce hepatotoxicity in humans [5, 6]. Earlier study has reported herbal extracts and their S9 metabolites‐derived from 90 medicinal plant species belonging to the Fabaceae, Asteraceae, and Lamiaceae families to induce mutagenic effects [7].

“Ayurveda” meaning the “science of life,” is a traditional Indian medicinal system that is well‐documented in classical textbooks [8–10]. The polyherbal immunomodulatory Ayurvedic medicine “Immunogrit” is formulated by blending of the extracts from *Pueraria tuberosa* (Roxb. ex Willd.) DC., *Polygonatum cirrhifolium* (Wall.) Royle, *Asparagus racemosus* Willd., *Roscoea procera* Wall., *Lilium polyphyllum* D. Don, *Habenaria intermedia* D. Don, *Dioscorea bulbifera* L., *Sida cordifolia* L., *Chlorophytum borivilianum* Santapau & R.R. Fern., *Mucuna pruriens* (L.) DC., and *Withania somnifera* (L.) Dunal extracts [8, 11]. Earlier toxicological evaluations of *P. tuberosa* (Roxb. ex Willd.) DC., *A. racemosus* Willd., *S. cordifolia* L., *M. pruriens* (L.) DC., and *W. somnifera* (L.) Dunal following the Organization for Economic Co‐operation and Development (OECD) guidelines had reported them to be safe [12–16]. However, *D. bulbifera* L. has been reported to cause liver toxicity through the cytochrome P450‐3A metabolism of its *cis*‐enediol intermediates [17]. *R. procera* Wall. is also reported to induce toxicity at a 5000 mg/kg body weight dose in Swiss albino mice [18].

Therefore, in this study, we had screened Immunogrit for repeated oral subacute toxicity in specific pathogen–free (SPF) male and female Sprague‐Dawley (SD) rats, following OECD 407 guidelines and Good Laboratory Practices compliance [19–21]. We also evaluated Immunogrit for latent toxicological responses in male and female SD rats for additional 14 days without exposure, post–28‐day stimulation at its highest tested dose. Induction of mortality, morbidity, and abnormality in clinical signs and symptoms, body weight, feed consumption habit, and functional and neurological behavior were routinely assessed throughout the study period. At the end of the study period, animals were analyzed for their terminal body and organ weights changes, as well as hematological, biochemical, and histopathological modifications. Additionally, the mutagenic potential of Immunogrit and its S9 metabolites was evaluated using the Ames test following OECD 471 guidelines in selected *Salmonella typhimurium* and *Escherichia coli* strains [22]. Phytochemical profile of Immunogrit was evaluated using ultraperformance liquid chromatography coupled with electrospray ionization quadrupole time‐of‐flight tandem mass spectrometer (UPLC/MS‐QToF). Finally, the “no‐observed‐adverse‐effect‐level” (NOAEL) for Immunogrit was determined for both male and female SD rats based on the study outcomes.

## 2. Materials and Methods

### 2.1. Test Article and Reagents

The test article Immunogrit (Batch No. 1DIG210037) was sourced from Divya Pharmacy (India) and stored at 24°C ± 3°C following the manufacturer’s instructions. Methylcellulose was obtained from Loba Chemie (India). Harris‐modified hematoxylin and eosin (H&E) stains, and standard phosphate‐buffered saline solution were purchased from Sigma‐Aldrich (USA). A Millipore Milli‐Q system (USA) distillation system was used to obtain deionized water. Liquid chromatography‐mass spectroscopy (LC‐MS) grade methanol, acetonitrile, and formic acid were procured from Honeywell GmbH (Germany). Dimethyl sulfoxide (DMSO) and bacteriological media were purchased from HiMedia Laboratories (India). Phenobarbital/5,6‐benzoflavone‐induced S9 fraction from SD rat liver (Cat No. 11–105.5), lyophilized NADPH REGENSYS (Cat No. 60–201.5L), and NADPH REGENSYS “A” (Cat No. 60–200.5) were purchased from Molecular Toxicology Inc. (USA). Standard mutagens were purchased from E. Merck (India).

### 2.2. UPLC/MS‐QToF Analysis

For the analysis, 100 mg of fine Immunogrit powder was suspended in 10 mL of (80:20) methanol:water solution. The suspension was sonicated for 30 min, followed by 5‐min centrifugation at 10000 rpm. Supernatant was filtered through a 0.22‐μm nylon filter, and the filtrate was collected for phytochemical profiling. A Xevo G2‐XS (Waters Corporation, USA) UPLC/MS‐QToF system coupled with the Acquity UPLCI class system (Waters Corporation, USA) and equipped with a positive and negative ionization mode electrospray ionization system was used in the study. Chromatographic separations of phytochemicals were done using the UPLC HSS T3 (100 × 2.1 mm, 1.8 μm) column at a temperature of 40°C, at a mobile phase of 0.3 mL/min flow rate. The gradient elution system consisted of 0.1% formic acid in water (Mobile Phase A) and 0.1% formic acid in acetonitrile (Mobile Phase B) adjusted to 20°C. The gradient program was based on the concentration of Mobile Phase B: 5%–10% B for 0–5 min, 10%–20% B for 5–15 min, 20%–30% B for 15–35 min, 30%–35% B for 35–45 min, 35%–80% B for 45–60 min, 80%–90% B for 60–65 min, 90%–5% B for 65–66 min, and 5% B for 66–70 min.

During analysis, 2 μL of the filtered test solutions was individually injected into the UPLC/MS‐QToF system, and chromatographs were recorded for 62 min [23]. Also, 200 pg/mL of leucine enkephalin (Waters Corporation, USA) was used as a reference control (lock mass: positive mode‐m/z 556.2766; negative mode‐m/z 554.2620) at a flow rate of 10 μL/min with 0.5‐s interval scans. A wide mass range (m/z 50–1200) was selected for acquisition of data in the MSe mode. The capillary and cone voltages, and source and desolvation temperatures were maintained at 1.0 kV, 120°C (positive mode) and 2.0 kV, 500°C (negative mode), respectively. High‐purity nitrogen gas was used for desolvation and cone with gas flow rates of 900 and 50 L/h. The low collision energy of 6 eV and high collision energy of 15–60 eV were applied in the collision cell for an accurate mass precursor and fragment ion data.

### 2.3. Animal Husbandry of SD Rats

A total of 36 SPF male SD rats with body weights 176.44–230.43 g, and 36 SPF nulliparous and nonpregnant female SD rats with body weights 160.53–193.87 g were purchased from Hylasco Bio‐Technology (India) Pvt. Ltd. (Telangana, India). All the animals were within the age range of 5–6 weeks upon arrival at the animal husbandry facility and subsequently quarantined for 6 days. The study was conducted in a vivarium registered with the Committee for Control and Supervision of Experiments on Animals (CCSEA), Ministry of Fisheries, Animal Husbandry and Dairying, Department of Animal Husbandry and Dairying, Government of India (Registration No.: 1784/PO/RcBiBt/S/2014/CPCSEA). The study protocol was reviewed and subsequently approved by the Institutional Animal Ethics Committee (IAEC) of Vanta Biosciences, India, vide Protocol Approval No.: 31/21. The animal husbandry and experimentations were performed in strict accordance with guidelines specified by the CCSEA. The study was reported following the ARRIVE guidelines [24].

Animals were housed in groups of three rats per polycarbonate cage. Ambience of the experiment room was maintained at a constant temperature of 20.1°C ± 4°C and 57%–69% relative humidity. Air supply in the experimental room was maintained at a minimum of 12 air changes/h. Light and dark cycles of 12 h each were maintained throughout the study period. Steam‐sterilized clean corn cob bedding material (Matha Agrotech, India) was provided to the animals and changed once a week or as required. Commercially available rodent specific pelleted feed (Krishna Valley Agrotech LLP, India) was provided to the animals during acclimatization and the study period. Reverse osmosis (RO) purified water was provided to animals *ad libitum* throughout the study period. A certified veterinarian monitored the animals’ health conditions.

### 2.4. Study Design and Test Article Dosing

Based on their body weights, male and female SD rats were randomly segregated into six groups (six animals per group), for each gender. The animal groups for each gender were assigned to four treatment (G1, G2, G3, and G4) groups, and two recovery (G1‐R and G4‐R) groups, a day before the initialization of the Immunogrit exposure. Freshly prepared methylcellulose solution (0.5% w/v) in RO water was mixed with triturated Immunogrit daily, immediately before oral gavage administration. Immunogrit was gavaged in SD rats for 28 consecutive days at daily doses of 100 (G2), 300 (G3), and 1000 (G4 and G4‐R) mg/kg body weight/day. The dose volume in all the animals was maintained at 10 mL/kg. Animals in the G1 and G1‐R (control) treatment groups received an equal volume of vehicle control (0.5% w/v methylcellulose solution) only. The recovery group animals were kept under observation without dosing for an additional 14 days.

### 2.5. Observation for Mortality, Morbidity, Clinical Signs, and Symptoms

Individual body weights were recorded on Days 1, 8, 15, 22, and 28 for all male and female animals and additionally on Days 35 and 42 for the G1‐R and G4‐R animal groups. Fasting body weights were taken before randomization, and on the necropsy day. Weekly feed consumption of the animal groups was recorded throughout the study period.

Animals were checked once daily for mortality, morbidity, and development of clinical signs and symptoms for the complete study duration. Detailed weekly clinical examination of the animals was done to check for changes in skin, fur, eyes, and mucous membranes and the presence of secretions or excretions.

### 2.6. Functional and Neurological Behavior Investigation

Functional and neurological behavior were recorded for the individual animal groups during the 4^th^ week and additionally during the 6^th^ week in the recovery groups. Any changes in body posture, activity, gait, vocalization, and clonic and tonic and involuntary movements were documented. Animals reactivity to handling, lachrymation, salivation, piloerection, palpebral closure, crusty eyes, exophthalmos and open field activity observations (ataxic gait, arousal, clonic and tonic convulsions, stereotype and bizarre behaviors, number of defecations/min, number of urine pools/min), stimulus reactivity observations (approach response, touch response, click response, tail pinch), pupil size, eye blink responses, forelimb response, hind limb extension, righting reflex on the ground, air righting reflex, catalepsy, hind limb extensor strength, hind limb landing foot splay), and neuromuscular observation (grip strength) were also recorded. The activity chamber was cleaned with a damp cloth and tissue paper after before and after animal examinations.

### 2.7. Ophthalmological and Clinical Examinations

Mydriasis was induced in the animal pupils by applying a few drops of 1% tropicamide (Batch No. 1B099, Aurolab, India) before the ophthalmic examination. Ophthalmic examinations of G1, G4, G1‐R, and G4‐R animal treatment groups were conducted during the acclimatization period and during the 4^th^ week for all the animal groups. Additional examination was conducted during the 6^th^ week for G1‐R and G4‐R animal groups.

### 2.8. Clinical Pathology Observations

Blood was drawn from the retro‐orbital sinus of animals under light anesthesia on terminal days for treatment and recovery animal groups, following overnight (12–14 h) fasting. Blood was collected in plain, 1.2 mg/mL EDTA‐K2/K3 containing, and 3.2% tri‐sodium citrate‐containing vials for hematological and biochemical analysis. Hematological parameters were analyzed using a hematology analyzer (ADVIA‐2120i, Siemens, USA). Differential leukocytes were counted following Giemsa staining using the AxioScope‐A1 brightfield microscope (Carl Zeiss IQS Deutschland GmbH, Germany). Reticulocytes were counted in the EDTA‐treated blood sample following methylene blue stain using the Zeiss AxioScope‐A1 brightfield microscope.

Blood drawn in 3.2% w/v sodium citrate‐containing vials were centrifuged (Sorvall Legend Micro 21R centrifuge, Thermo Scientific, USA) at 3000 × *g* for 10 min at 4°C for isolating plasma and measuring clotting time. Prothrombin time (sec), and activated partial thromboplastin time (sec) were analyzed in plasma using a coagulation analyzer (Fourclot Analyzer, Robonik (India) Pvt. Ltd., India).

Blood without anticoagulant was left undisturbed at room temperature for 1 h and then centrifuged in a refrigerated centrifuge at 3000 × *g* for 15 min at 4°C for the separation of serum. Biochemical parameters analyzed in the separated serum samples were total bilirubin (T. Bil [mg/dL]), cholesterol (CHOL [mg/dL]), calcium (Ca [mg/dL]), urea [mg/dL], aspartate aminotransferase (AST [U/L]), alkaline phosphatase (ALP [U/L]), triglycerides (TGL [mg/dL]), glucose (Glu [mg/dL]), phosphate (Phos [mg/dL]), alanine aminotransferase (ALT [U/L]), creatinine (Creat [mg/dL]), albumin (Alb [g/dL]), total protein (T. Pro [g/dL]), blood urea nitrogen (BUN [mg/dL]), globulin (Glob [g/dL]), sodium (Na [mmol/L]), potassium (K [mmol/L]), chloride (Cl [mmol/L]), and direct bilirubin (D. Bil [mg/dL]) using an autoanalyzer (Cobas C111, Roche Diagnostics, USA).

### 2.9. Urine Biochemical and Cytological Analysis

Urine was collected in thymol blue crystals containing beakers from animals placed in metabolic cages on Days 28 and 42. During urine collection, animals were fasted and provided water *ad libitum*. Collected urine samples were analyzed for volume (mL), bilirubin content (Bil [mg/dL]), ketone content (mg/dL), specific gravity (SG), red blood corpuscles (RBCs/μL), pH condition, protein content (mg/dL), urobilinogen content (mg/dL), glucose content (mg/dL), white blood cells (WBCs/μL), and nitrite (mg/dL) using an urometer (Urometer 720, USA).

### 2.10. Necropsy and Gross Pathology

Twelve‐hour fasted animals were sacrificed using thiopentone overdose. During the fasting period, animals had free access to drinking water *ad libitum.* Gross inspection of the exterior body surface, orifices, and cranial, thoracic, abdominal cavity, and their contents were observed in all animals. Organs were harvested, and adherent fatty tissues were carefully trimmed following gross pathology examination. Wet weights of the vital and secondary organs were recorded for all animals, and relative organ weights were calculated using the following equation:

(1) relative organ weights g=organ weight gterminal body weight g×100.

### 2.11. Histopathological Analysis

Testes, eyes, Harderian gland, and optic nerve were harvested from the euthanized animals and fixed in modified Davidson’s fixative for 24 h. The tissue samples were later transferred into a 10% neutral buffered formalin. Other harvested organs were directly fixed in a 10% neutral buffered formalin solution and embedded in paraffin wax. Using a steel blade microtome (RM 2245, Leica Biosystems Nussloch GmbH, Germany), 5‐μm‐thick tissue sections were dissected and stained with H&E stain. The H&E‐stained tissue samples were analyzed blindly by a veterinarian histopathologist using a Zeiss AxioScope‐A1 brightfield microscope. Representative images were acquired for each tissue section for all the animal groups and processed using imaging software.

### 2.12. Bacterial Mutagenicity (Ames) Assay

*S. typhimurium* tester strains TA98 (BAA 2720), TA100 (BAA 2721), TA1537 (29630), TA1535 (29629), and *E. coli* tester strain WP2uvrA (49979) were obtained from the American Type Culture Collection (USA). The bacterial strains were cultured overnight in nutrient broth media at 37°C. The S9 metabolic mix consisting of 10% S9 fraction supplemented with glucose‐6‐phosphate, NADPH, MgCl_2_/KCl, and 0.1 M phosphate buffer (pH 7.4) was prepared following OECD 471 guidelines [22, 25, 26]. Mutagenicity of Immunogrit was evaluated using the plate incorporation method in the absence of metabolic activation and following preincubation in S9 metabolic mix.

Half‐log dilutions of Immunogrit and standard mutagens 4‐nitroquinoline, sodium azide, 4‐nitroquinoline, and 2‐aminoanthracene were prepared in DMSO solution. DMSO alone was used as the vehicle control. Standard mutagens 4‐nitroquinoline (0.15 μg/plate) for TA98, sodium azide (0.5 μg/plate) for TA100 and TA1535, 9‐aminoacridine (50 μg/plate) for TA1537, and 4‐nitroquinoline (0.5 μg/plate) for *E. coli* WP2uvrA were used as positive controls in the absence of S9 metabolic activation, while 2‐aminoanthracene (20 μg/plate) was applied in all strains with S9 metabolic activation.

For the assay, 100 μL of test bacterial cultures (1‐2 × 10^9^ cells/mL) was incubated at 37°C with different concentrations of Immunogrit or standard mutagens in the absence or presence of S9 mix in a tube for 30 min, without shaking [22]. Subsequently, the bacterial suspensions were mixed with 2 mL of soft agar containing 0.6% bacto‐agar, 0.5% NaCl, and 50 μM biotin and histidine for *S. typhimurium* strains, and 50 μM tryptophan for the *E. coli* strain) and poured onto a minimal agar plate (1.5% bacto‐agar, Vogel‐Bonner E medium, 0.4% glucose), and incubated at 37°C for 64–72 h. Revertant colonies were counted visually, and the mutagenicity ratio (MR) was calculated for each tested condition against the vehicle control (DMSO). Test article with an MR of 2.0 and above was considered positive for inducing mutagenicity.

### 2.13. Statistical Analysis

The results are presented as mean ± standard deviation. Statistical analysis was performed using GraphPad Prism Version 7.04 (GraphPad Software, USA) software. Body weight and feed consumption changes were analyzed using the two‐way analysis of variance (ANOVA) followed by Tukey’s multiple comparison post hoc test. Motor activity, landing foot splay, hematology, clinical chemistry, and organ weight data were analyzed using the one‐way ANOVA followed by Dunnett’s multiple comparisons post hoc test. In recovery groups, a *t* test was applied to all homogeneous data, whereas heterogeneous data were analyzed using Mann–Whitney’s test. Student’s *t* test was performed to assess the statistical significance of the study. *p* value < 0.05 was considered statistically significant.

## 3. Results

**FIGURE 1:**
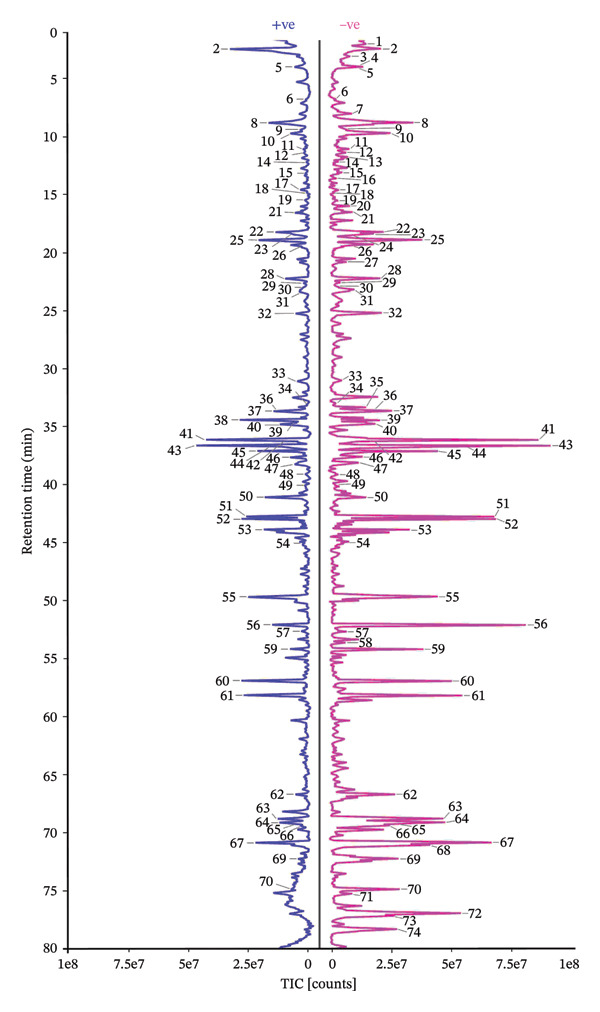
Ultraperformance liquid chromatography coupled with electrospray ionization quadrupole time‐of‐flight tandem mass (UPLC/MS‐QToF) spectrometry analysis of Immunogrit. Overlay chromatograms of UPLC/MS‐QToF analysis for Immunogrit (Batch No. 1DIG210037) were performed in positive and negative modes. Phytochemical analysis of their total ion chromatogram (TIC) and mass‐to‐charge ratio identified 74 compounds in Immunogrit. Peak numbers are assigned to identified phytochemicals by the detailed spectrometric data shown in Table 1.

A comprehensive UPLC/MS‐QToF analysis of the Immunogrit identified 74 phytochemicals based on their mass‐to‐charge ratio including phenolic acids (gallic acid, protocatechuic acid, 3‐hydroxy‐5‐methoxybenzoic acid, ellagic acid, and 3‐O‐methylellagic acid‐3′‐O‐α‐L‐rhamnopyranoside), flavonoids (flavonols: quercetin, kaempferol, rutin, isoquercetin, kaempferol‐3‐gentiobioside, isorhamnetin‐3‐O‐β‐gentiobioside, kaempferol‐3‐O‐rutinoside, isorhamnetin 3‐O‐beta‐glucopyranoside‐7‐O‐alpha‐rhamnopyranoside, astragalin, isorhamnetin‐3‐rutinoside, isorhamnetin 3‐O‐(6‐O‐*p*‐coumaroyl)‐glucoside, kaempferide, isokaempferide, and tiliroside; flavones: apigenin 7‐O‐beta‐D‐glucopyranoside, luteolin, apigenin 7‐rutinoside, and irisolidone; flavanone: diosmin; and flavan‐3‐ols (catechins): scopoletin, catechin, epicatechin, (+)‐catechin‐5‐O‐beta‐D‐glucopyranoside, catechin 7‐O‐β‐D‐glucopyranoside, epicatechin 5‐O‐β‐D‐glucopyranoside, and gallocatechin); proanthocyanidins (procyanidins B1, B2, B3, B4, C1, C2, and arecatannin A1 and B1); lignans (lariciresinol dimethyl ether, and stilbenes [resveratroloside]); coumarin derivatives (5,7‐dihydroxy‐8‐(3‐methylbut‐2‐enyl)‐6‐(2‐methylpropanoyl)‐4‐phenylchromen‐2‐one, 5,7‐dihydroxy‐8‐isobutyryl‐6‐(3‐methyl‐2‐buten‐1‐yl)‐4‐phenyl‐2H‐chromen‐2‐one, and menisdaurin B); saponins (anemarsaponin F, shatavarin IX, agaveside B, asparacoside, asparagoside F and G, asparanin B9, tigogenin pentasaccharide, gitonin, achyranthoside II, trigofoenoside F, balanitoside, tribulosin, puerarin 4′‐O‐glucoside, kaikasaponin II); and triterpenoids ((3β)‐olean‐12‐en‐3‐yl β‐D‐galactopyranosyl‐(1–2)‐[β‐D‐xylopyranosyl‐(1–3)]‐β‐D‐glucopyranosiduronic and pseudolaric acid A) (Figure 1, Table 1). Other compounds identified in Immunogrit included coumarin glycoside pectachol, curcuminoid hexahydrocurcumin, glycyrdione B, glyinflanin C, gancaonin Q, 3‐O‐acetylepisamarcandin, ganolactone, lucidenolactone, and flemiphyllin (Figure 1, Table 1).

**TABLE 1:** Phytochemical profiling of Immunogrit.

Peak ID	RT (min)	Observed m/z	Adducts	Neutral mass (Da)	Expected m/z	Mass error (mDa)	Formula	Component name
1	2.01	169.0145	−H	170.0215	169.0142	0.2	C_7_H_6_O_5_	Gallic acid

2	2.50	328.1376	+H	327.1318	328.1391	−1.5	C_15_H_21_NO_7_	Menisdaurin B
	2.45	326.1253	−H		326.1245	0.8		

3	3.04	451.1248	−H	452.1319	451.1246	0.2	C_21_H_24_O_11_	(+)‐Catechin‐5‐O‐beta‐D‐glucopyranoside

4	3.94	153.0193	−H	154.0266	153.0193	0.0	C_7_H_6_O_4_	Protocatechuic acid

5	4.01	453.1384	+H	452.1319	453.1392	−0.8	C_21_H_24_O_11_	Catechin 7‐O‐β‐D‐glucopyranoside
	3.98	451.1249	−H		451.1246	0.3		

6	6.79	453.1388	+H	452.1319	453.1392	−0.4	C_21_H_24_O_11_	Epicatechin 5‐O‐β‐D‐glucopyranoside
	6.74	451.1245	−H		451.1246	−0.1		

7	7.99	237.0407	+HCOO	192.0423	237.0405	0.3	C_10_H_8_O_4_	Scopoletin

8	8.84	579.1499	+H	578.1424	579.1497	0.2	C_30_H_26_O_12_	Procyanidin B1
	8.80	577.1356	−H		577.1351	0.5		

9	9.39	579.1498	+H	578.1424	579.1497	0.1	C_30_H_26_O_12_	Procyanidin B2
	9.35	577.1353	−H		577.1351	0.2		

10	9.71	291.0851	+H	290.0790	291.0863	−1.2	C_15_H_14_O_6_	Catechin
	9.67	289.0724	−H		289.0717	0.6		

11	11.07	867.2151	+H	866.2058	867.2131	2.0	C_45_H_38_O_18_	Procyanidin C1
	11.04	865.2009	−H		865.1985	2.4		

12	11.44	867.2157	+H	866.2058	867.2131	2.6	C_45_H_38_O_18_	Procyanidin C2
	11.40	865.2018	−H		865.1985	3.3		

13	11.94	305.0704	−H	306.0740	305.0667	3.7	C_15_H_14_O_7_	Gallocatechin

14	12.13	579.1509	+H	578.1424	579.1497	1.2	C_30_H_26_O_12_	Procyanidin B3
	12.10	577.1349	−H		577.1351	−0.2		

15	13.12	291.0850	+H	290.0790	291.0863	−1.3	C_15_H_14_O_6_	Epicatechin
	13.08	289.0725	−H		289.0717	0.7		

16	13.50	167.0350	−H	168.0423	167.0350	0.0	C_8_H_8_O_4_	3‐Hydroxy‐5‐methoxybenzoic acid

17	14.64	867.2154	+H	866.2058	867.2131	2.3	C_45_H_38_O_18_	Arecatannin A1
	14.60	865.2015	−H		865.1985	3.0		

18	14.86	867.2146	+H	866.2058	867.2131	1.6	C_45_H_38_O_18_	Arecatannin B1
	14.83	865.2015	−H		865.1985	2.9		

19	15.51	579.1511	+H	578.1424	579.1497	1.4	C_30_H_26_O_12_	Procyanidin B4
	15.48	577.1353	−H		577.1351	0.2		

20	15.97	389.1249	−H	390.1315	389.1242	0.7	C_20_H_22_O_8_	Resveratroloside

21	16.49	449.1076	+H	448.1006	449.1079	−0.3	C_21_H_20_O_11_	Quercitrin
	16.38	447.0941	−H		447.0933	0.8		

22	18.23	433.1123	+H	432.1057	433.1130	−0.6	C_21_H_20_O_10_	Apigenin 7‐O‐beta‐D‐glucopyranoside
	18.20	431.0991	−H		431.0984	0.7		

23	18.40	611.1616	+H	610.1534	611.1607	0.9	C_27_H_30_O_16_	Rutin
	18.36	609.1468	−H		609.1461	0.7		

24	18.53	300.9997	−H	302.0063	300.9990	0.7	C_14_H_6_O_8_	Ellagic acid

25	18.88	611.1607	+H	610.1534	611.1607	0.1	C_27_H_30_O_16_	Kaempferol‐3‐gentiobioside
	18.85	609.1468	−H		609.1461	0.7		

26	19.54	465.1023	+H	464.0955	465.1028	−0.5	C_21_H_20_O_12_	Isoquercetin
	19.50	463.0886	−H		463.0882	0.4		

27	20.79	639.1571	−H	640.1640	639.1567	0.4	C_28_H_32_O_17_	Isorhamnetin‐3‐O‐β‐gentiobioside

28	22.24	595.1663	+H	594.1585	595.1658	0.6	C_27_H_30_O_15_	Kaempferol‐3‐O‐rutinoside
	22.19	593.1519	−H		593.1512	0.7		

29	22.60	625.1777	+H	624.1690	625.1763	1.4	C_28_H_32_O_16_	Isorhamnetin 3‐O‐beta‐glucopyranoside‐7‐O‐alpha‐rhamnopyranoside
	22.56	623.1622	−H		623.1617	0.4		

30	23.01	449.1063	+H	448.1006	449.1079	−1.5	C_21_H_20_O_11_	Astragalin
	22.95	447.0932	−H		447.0933	−0.1		

31	23.38	625.1790	+H	624.1690	625.1763	2.7	C_28_H_32_O_16_	Isorhamnetin‐3‐rutinoside
	23.34	623.1622	−H		623.1617	0.4		

32	25.24	463.0868	+H	462.0798	463.0871	−0.3	C_21_H_18_O_12_	3‐O‐Methylellagic acid‐3′‐O‐α‐L‐rhamnopyranoside
	25.19	461.0727	−H		461.0725	0.1		

33	31.07	631.1656	+Na	608.1741	631.1633	2.2	C_28_H_32_O_15_	Diosmin
	31.04	653.1731	+HCOO		653.1723	0.8		

34	33.09	303.0489	+H	302.0427	303.0500	−1.1	C_15_H_10_O_7_	Quercetin
	33.01	301.0360	−H		301.0354	0.7		

35	33.33	1095.5292	+HCOO	1050.5247	1095.5229	6.3	C_50_H_82_O_23_	Anemarsaponin F

36	33.60	287.0548	+H	286.0477	287.0550	−0.2	C_15_H_10_O_6_	Luteolin
	33.54	285.0412	−H		285.0404	0.8		

37	33.65	711.2147	+H	710.2058	711.2131	1.6	C_32_H_38_O_18_	7‐[[6‐O‐(5‐O‐D‐Apio‐beta‐D‐furanosyl‐D‐apio‐beta‐D‐furanosyl)‐beta‐D‐glucopyranosyl]oxy]‐4′‐methoxy‐
	33.62	755.2053	+HCOO		755.2040	1.3		

38	34.41	1167.578	+H	1166.5720	1167.5793	−1.3	C_55_H_90_O_26_	Tigogenin pentasaccharide

39	34.61	1073.5151	+Na	1050.5247	1073.5139	1.2	C_50_H_82_O_23_	Gitonin
	34.62	1095.5292	+HCOO		1095.5229	6.3		

40	34.79	579.1711	+H	578.1636	579.1709	0.3	C_27_H_30_O_14_	Puerarin 4′‐O‐glucoside
	34.76	623.1622	+HCOO		623.1618	0.4		

41	36.12	1105.5432	+Na	1082.5509	1105.5401	3.1	C_51_H_86_O_24_	Asparagoside G
	36.14	1127.5563	+HCOO		1127.5491	7.2		

42	36.35	601.1544	+Na	578.1636	601.1528	1.6	C_27_H_30_O_14_	Apigenin 7‐rutinoside
	36.32	623.1632	+HCOO		623.1618	1.4		

43	36.61	1075.5310	+Na	1052.5403	1075.5295	1.4	C_50_H_84_O_23_	Asparanin B9
	36.63	1097.5451	+HCOO		1097.5385	6.6		

44	36.76	595.1460	+H	594.1373	595.1446	1.3	C_30_H_26_O_13_	Tiliroside
	36.73	593.1309	−H		593.1300	0.8		

45	37.08	943.4889	+Na	920.4981	943.4873	1.6	C_45_H_76_O_19_	Asparagoside E
	37.10	965.5000	+HCOO		965.4963	3.7		

46	37.61	625.1570	+H	624.1479	625.1552	1.8	C_31_H_28_O_14_	Isorhamnetin 3‐O‐(6‐O‐*p*‐coumaroyl)‐glucoside
	37.58	623.1415	−H		623.1406	0.9		

47	38.26	301.0695	+H	300.0634	301.0707	−1.2	C_16_H_12_O_6_	Kaempferide
	38.21	299.0567	−H		299.0561	0.6		

48	39.04	287.0540	+H	286.0477	287.0550	−1.0	C_15_H_10_O_6_	Kaempferol
	38.98	285.0410	−H		285.0404	0.5		

49	40.03	301.0700	+H	300.0634	301.0707	−0.7	C_16_H_12_O_6_	Isokaempferide
	39.98	299.0567	−H		299.0561	0.6		

50	41.07	1167.5826	+H	1166.5720	1167.5793	3.3	C_55_H_90_O_26_	Agaveside B
	41.09	1165.5722	−H		1165.5647	7.4		

51	42.74	1065.5471	+H	1064.5403	1065.5476	−0.5	C_51_H_84_O_23_	Trigofoenoside F
	42.76	1109.5441	+HCOO		1109.5385	5.6		

52	42.94	1035.5378	+H	1034.5298	1035.5371	0.7	C_50_H_82_O_22_	Asparagoside F
	42.97	1079.5342	+HCOO		1079.5280	6.2		

53	43.85	903.4974	+H	902.4875	903.4948	2.6	C_45_H_74_O_18_	Shatavarin IX
	43.97	947.4899	+HCOO		947.4857	4.2		

54	45.12	315.0851	+H	314.0790	315.0863	−1.2	C_17_H_14_O_6_	Irisolidone
	45.08	313.0723	−H		313.0717	0.6		

55	49.68	285.0746	+H	284.0685	285.0758	−1.1	C_16_H_12_O_5_	Biochanin A
	49.63	283.0617	−H		283.0612	0.5		

56	52.06	949.5170	+Na	926.5239	949.5131	3.8	C_48_H_78_O_17_	Kaikasaponin II
	52.09	971.5271	+HCOO		971.5221	5.0		

57	52.61	1027.5116	+Na	1004.5192	1027.5084	3.2	C_49_H_80_O_21_	Asparacoside
	52.65	1049.5229	+HCOO		1049.5174	5.5		

58	53.61	779.4604	−H	780.4660	779.4587	1.7	C_42_H_68_O_13_	Achyranthoside II

59	54.14	919.5052	+Na	896.5133	919.5025	2.7	C_47_H_76_O_16_	(3β)‐Olean‐12‐en‐3‐yl β‐D‐galactopyranosyl‐(1–2)‐[β‐D‐xylopyranosyl‐(1–3)]‐β‐D‐glucopyranosiduronic
	54.17	895.5092	−H		895.5060	3.2		

60	56.88	1087.5350	+Na	1064.5403	1087.5295	5.4	C_51_H_84_O_23_	Balanitoside
	56.91	1109.5438	+HCOO		1109.5385	5.2		

61	58.14	1173.5707	+Na	1150.5771	1173.5663	4.3	C_55_H_90_O_25_	Tribulosin
	58.17	1195.5813	+HCOO		1195.5753	6.0		

62	66.69	375.1794	+H	374.1729	375.1802	−0.8	C_21_H_26_O_6_	Hexahydrocurcumin
	66.67	373.1659	−H		373.1656	0.2		

63	68.78	393.1691	+H	392.1624	393.1697	−0.6	C_24_H_24_O_5_	5,7‐Dihydroxy‐8‐(3‐methylbut‐2‐enyl)‐6‐(2‐methylpropanoyl)‐4‐phenylchromen‐2‐one
	68.77	391.1554	−H		391.1551	0.3		

64	69.11	389.1955	+H	388.1886	389.1959	−0.4	C_22_H_28_O_6_	Pseudolaric acid A
	69.08	387.1817	−H		387.1813	0.4		

65	69.26	393.1698	+H	392.1624	393.1697	0.2	C_24_H_24_O_5_	5,7‐Dihydroxy‐8‐isobutyryl‐6‐(3‐methyl‐2‐buten‐1‐yl)‐4‐phenyl‐2H‐chromen‐2‐one
	69.25	391.1555	−H		391.1551	0.4		

66	69.42	389.1962	+H	388.1886	389.1959	0.3	C_22_H_28_O_6_	Lariciresinol dimethyl ether
	69.39	387.1817	−H		387.1813	0.4		

67	70.85	407.1852	+H	406.1780	407.1853	−0.1	C_25_H_26_O_5_	Glycyrdione B
	70.83	405.1707	−H		405.1707	−0.1		

68	71.06	405.1706	−H	406.1780	405.1707	−0.2	C_25_H_26_O_5_	Glyinflanin C

69	72.24	407.1848	+H	406.1780	407.1853	−0.5	C_25_H_26_O_5_	Gancaonin Q
	72.23	405.1708	−H		405.1707	0.0		

70	74.88	443.2441	+H	442.2355	443.2428	1.2	C_26_H_34_O_6_	3‐O‐Acetylepisamarcandin
	74.86	441.2285	−H		441.2282	0.2		

71	75.26	441.2286	−H	442.2355	441.2282	0.3	C_26_H_34_O_6_	Pectachol
72	76.92	455.2437	−H	456.2512	455.2439	−0.2	C_27_H_36_O_6_	Ganolactone

73	77.16	455.2439	−H	456.2512	455.2439	0.0	C_27_H_36_O_6_	Lucidenolactone

74	78.33	475.2485	+H	474.2406	475.2479	0.6	C_30_H_34_O_5_	Flemiphyllin
	78.29	473.2336	−H		473.2333	0.3		

*Note:* Using ultraperformance liquid chromatography coupled with electrospray ionization quadrupole time‐of‐flight tandem mass (UPLC/MS‐QToF) spectrometry technique, 74 phytochemicals were identified based on the mass (m)‐to‐charge (z) ratio in the Immunogrit formulation. Peak identification on the representative chromatograms is depicted in Figure 1.

**FIGURE 2:**
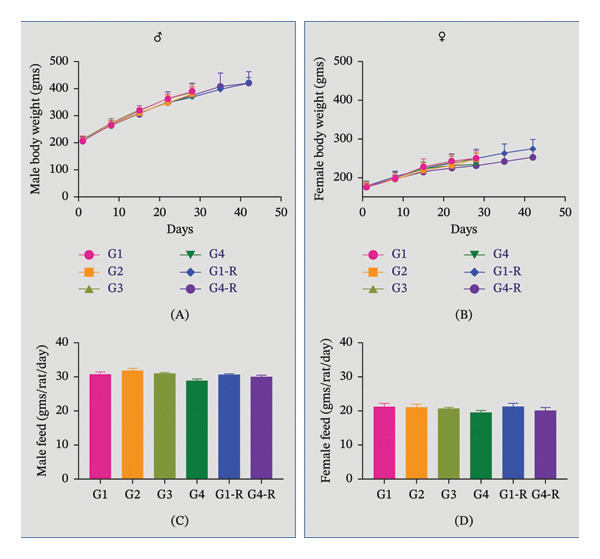
Body weight and feeding habit analysis in Immunogrit‐exposed male and female Sprague‐Dawley rats. Body weights of the (A) male and (B) female Sprague‐Dawley rats were recorded individually for the treatment group vehicle control (G1), 100 mg/kg body weight (G2), 300 mg/kg body weight (G3), 1000 mg/kg body weight (G4), recovery group vehicle control (G1‐R), and 1000 mg/kg body weight (G4‐R) animals on Days 1, 8, 15, 22, and 28, and additionally on Days 35 and 42 for G1‐R and G4‐R animals. Weekly feed consumption of (C) male and (D) female Sprague‐Dawley rats was recorded during the treatment and recovery phases. Results are presented as mean ± standard deviation. Statistical analysis was performed using a two‐way analysis of variance (ANOVA) followed by Tukey’s multiple comparison post hoc test.

Repeated oral gavage administration of Immunogrit in male and female SD rats did not induce mortality, morbidity, development of clinical signs, or ophthalmic defects up to the highest tested dose of 1000 mg/kg body weight/day, throughout the study period (Table 2). Also, no changes associated with body weights or feeding habits were observed in the Immunogrit‐exposed animals (Figure 2A, B, C, D). No latent noxious effects in terms of mortality, body weight change, clinical signs, ophthalmic defects, and feeding habits were observed in the male and female SD rats belonging to the G1‐R and G4‐R groups (Figure 2A, B, C, D; Table 2).

**TABLE 2:** Mortality, morbidity, and clinical signs analysis in Immunogrit‐exposed male and female SD rats.

Groups		Immunogrit doses (mg/kg body weight/day)	Mortality/no. of animals	Clinical signs	Detailed clinical signs	Ophthalmoscopy observation (before treatment)	Ophthalmoscopy observation (after treatment)
Male rats (♂)	G1	0	0/6	NAD	NAD	NAD	NAD
	G2	100	0/6	NAD	NAD	NAD	NAD
	G3	300	0/6	NAD	NAD	NAD	NAD
	G4	1000	0/6	NAD	NAD	NAD	NAD
	G1‐R	0	0/6	NAD	NAD	NAD	NAD
	G4‐R	1000	0/6	NAD	NAD	NAD	NAD

Female rats (♀)	G1	0	0/6	NAD	NAD	NAD	NAD
	G2	100	0/6	NAD	NAD	NAD	NAD
	G3	300	0/6	NAD	NAD	NAD	NAD
	G4	1000	0/6	NAD	NAD	NAD	NAD
	G1‐R	0	0/6	NAD	NAD	NAD	NAD
	G4‐R	1000	0/6	NAD	NAD	NAD	NAD

*Note:* Both the genders of Sprague‐Dawley rats in G1, G2, G3, G4, G1‐R, and G4‐R groups during the 28‐day administration period with Immunogrit and 14‐day recovery period were kept under constant observation for mortality, and development of morbidity and clinical signs by a trained veterinarian.

Abbreviation: NAD, no abnormality detected.

All animals showed normal physical activities including random sitting, standing, and sleeping postures independent of the Immunogrit stimulation conditions (G1, G2, G3, G4, G1‐R, and G4‐R) throughout the study period (Table 3). The Immunogrit‐exposed animal groups also showed normal autonomic activities including vocalization, ataxic gait, and involuntary clonic and tonic convulsions (Table 3). They appeared to show no resistance to physical handling. All the animal groups showed signs of lachrymation, salivation, piloerection, palpebral closure, crusty eyes, or exophthalmos, independent of Immunogrit exposure conditions (Table 3). Similarly, no latent development of behavioral and autonomic abnormalities was observed in the G1‐R and G4‐R group animals during the 14‐day recovery period.

**TABLE 3:** Functional and neurological behavior assessments in Immunogrit‐exposed male and female Sprague‐Dawley rats.

**Immunogrit dose (mg/kg body weight/day)**	**0**	**100**	**300**	**1000**	**0**	**1000**
**Male rats (♂)**	**G1**	**G2**	**G3**	**G4**	**G1-R**	**G4-R**
Body posture						
Sitting or standing normally (S)	6	6	1	4	6	6
Asleep, lying on side or curled up (A)	0	0	3	0	0	0
Rearing (R)	0	0	2	2	0	0
Active animal	6	6	6	6	6	6
Gait (NAD)	6	6	6	6	6	6
Presence of vocalization	0	0	0	0	0	0
Reactivity to handling low (no resistance, rat is easy to handle)	6	6	6	6	6	6
Presence of lachrymation	0	0	0	0	0	0
Presence of salivation	0	0	0	0	0	0
Presence of piloerection	0	0	0	0	0	0
Presence of palpebral closure	0	0	0	0	0	0
Presence of crusty eyes	0	0	0	0	0	0
Presence of exophthalmos	0	0	0	0	0	0
Body temperature (^o^F) max–min	98.6 to 97.2	98.9 to 97.4	99.4 to 98.3	99.1 to 97.5	98.6 to 97.8	98.8 to 97.6

*Open field observations*						
Presence of ataxic gait	0	0	0	0	0	0
Arousal						
Normal	6	6	6	6	6	6
High	0	0	0	0	0	0
Presence of clonic convulsions	0	0	0	0	0	0
Presence of tonic convulsions	0	0	0	0	0	0
Presence of stereotyped behavior	0	0	0	0	0	0

**Female rats (♀)**	**G1**	**G2**	**G3**	**G4**	**G1-R**	**G4-R**

Body posture body posture						
Sitting or standing normally (S)	3	2	2	1	4	3
Asleep, lying on side or curled up (A)	0	0	0	0	0	0
Rearing (R)	3	4	4	5	2	3
Active animal	6	6	6	6	6	6
Gait (NAD)	6	6	6	6	6	6
Presence of vocalization	0	0	0	0	0	0
Reactivity to handling low (no resistance, rat is easy to handle)	6	6	6	6	6	6
Presence of lachrymation	0	0	0	0	0	0
Presence of salivation	0	0	0	0	0	0
Presence of piloerection	0	0	0	0	0	0
Presence of palpebral closure	0	0	0	0	0	0
Presence of crusty eyes	0	0	0	0	0	0
Presence of exophthalmos	0	0	0	0	0	0
Body temperature (^o^F) max–min	99.1 to 97.9	98.7 to 97.6	99.7 to 97.8	99.4 to 97.9	98.7 to 97.9	98.1 to 96.4

*Open field observations*						
Presence of ataxic gait	0	0	0	0	0	0
Arousal						
Normal	6	6	6	6	6	6
High	0	0	0	0	0	0
Presence of clonic convulsions	0	0	0	0	0	0
Presence of tonic convulsions	0	0	0	0	0	0
Presence of stereotyped behavior	0	0	0	0	0	0

*Note:* Both the genders of Sprague‐Dawley rats in G1, G2, G3, G4, G1‐R, and G4‐R groups during the 28‐day treatment period of Immunogrit and the 14‐day recovery period were evaluated for functional, neurological, and behavioral changes during and post‐Immunogrit treatment by a trained veterinarian throughout the study period. Results represent the number of animals per treatment group for each observed parameter.

Abbreviation: NAD, no abnormality detected**.**

**FIGURE 3:**
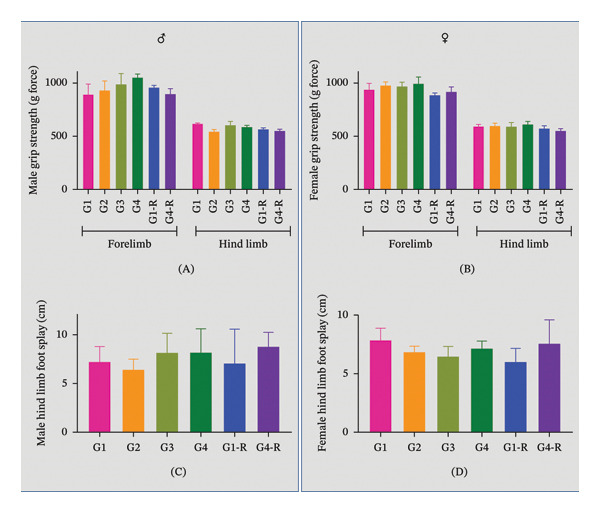
Foot grip strength and hind limb landing foot splay analysis in Immunogrit‐exposed male and female Sprague‐Dawley rats. Fore and hind limb foot grip strength analysis of (A) male and (B) female Sprague‐Dawley rats was recorded individually for the treatment group vehicle control (G1), 100 mg/kg body weight (G2), 300 mg/kg body weight (G3), 1000 mg/kg body weight (G4), recovery group vehicle control (G1‐R), and 1000 mg/kg body weight (G4‐R) animals during the 4^th^ week and additionally during 6^th^ week for G1‐R and G4‐R animals. Similarly, hind limb landing foot splay was recorded for the (C) male and (D) female Sprague‐Dawley rats during the 4^th^ and 6^th^ weeks, respectively, based on the termination of the subacute Immunogrit treatment and recovery phases. Results are presented as mean ± standard deviation. Statistical analysis was performed using a one‐way ANOVA followed by Dunnett’s multiple comparisons post hoc test. In recovery groups, a *t* test was applied to all homogeneous data, whereas heterogeneous data were analyzed using Mann–Whitney’s test. *p* value < 0.05 was considered statistically significant.

Body temperature of the male and female animal groups appeared normal (average body temperature of 98.27 ± 0.74 °F) during the 28‐day Immunogrit exposure and 14‐day recovery periods (Table 3). The Immunogrit‐exposed animal’s forelimb and hind limb grip strength, and hind limb landing foot splays remained unaltered throughout the study period (Figure 3A, B, C, D). These observations confirmed that Immunogrit did not induce any immediate or latent physiological or neurological defects in male and female SD rats throughout the study period.

**FIGURE 4:**
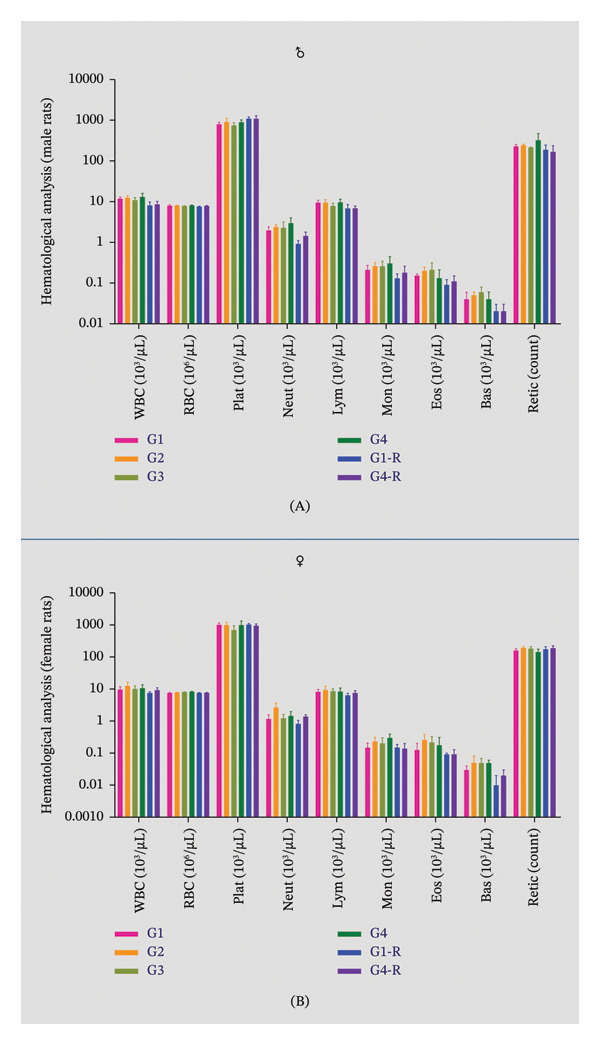
Differential leukocyte and reticulocyte counts in Immunogrit‐exposed male and female Sprague‐Dawley rats. Differential leukocyte (white blood cells [WBCs], red blood corpuscles [RBCs], platelets [Plat], neutrophils [Neut], lymphocytes [Lym], monocytes [Mon], eosinophils [Eos], and basophils [Bas]) and reticulocyte (Retic) counts were recorded in blood drawn from (A) male and (B) female Sprague‐Dawley rats at the end of the treatment and recovery phases following overnight (12–14 h) fasting. Results are presented as mean ± standard deviation. Statistical analysis was performed using a one‐way ANOVA followed by Dunnett’s multiple comparisons post hoc test. In recovery groups, a *t* test was applied to all homogeneous data, whereas heterogeneous data were analyzed using Mann–Whitney’s test. *p* value < 0.05 was considered statistically significant.

Detailed hematological analysis in both the SD rat genders revealed no clinically relevant alterations during the 28‐day Immunogrit exposure and the 14‐day recovery periods. Differential blood count analysis showed a steady hematological profile for WBCs, red blood cells (RBCs), platelets, neutrophils, lymphocytes, monocytes, eosinophils, and basophils throughout the study period, across all the Immunogrit exposure conditions (Figure 4A, B). No variations were observed in the reticulocyte count obtained from male and female SD rats undergoing 28‐day Immunogrit exposure or during the 14‐day recovery phases (Figure 4A, B). Notably, the average hemoglobin content (18.89 ± 0.32 g/dL), hematocrit percentage (46.63 ± 1.54%), mean corpuscular volume (59.79 ± 1.41 fL), mean corpuscular hemoglobin (24.24 ± 0.58 pg) content and (40.54 ± 1.06 g/dL) concentration, and mean platelet volume (6.71 ± 0.27 fL) present in all animal groups represented the absence of any hematological abnormalities associated with Immunogrit exposure regimen (Table 4). The coagulation cascade in both the genders of rats appeared unaffected by the Immunogrit exposure, as observed through the consistent levels of average prothrombin time (23.94 ± 1.31 s) and activated partial thromboplastin (25.78 ± 1.31 s) values (Table 4). Finally, all the reported hematological values were observed to be within the historical reference range reported within the study center and elsewhere [27–29].

**TABLE 4:** Hematological profile in Immunogrit‐exposed male and female Sprague‐Dawley rats.

Male rats (♂)	Hb (g/dL)	HCT (%)	MCV (fL)	MCH (pg)	MCHC (g/dL)	MPV (fL)	PT (sec)	APTT (sec)
G1	19.13 ± 0.25	49.28 ± 0.97	62.3 ± 2.68	24.23 ± 1.19	38.87 ± 0.55	6.88 ± 0.39	23.57 ± 2.9	26.93 ± 3.12
G2	18.58 ± 0.36	47.63 ± 1.52	60.4 ± 1.62	23.58 ± 0.65	39.05 ± 1.2	6.88 ± 0.33	26.27 ± 1.83	25.13 ± 2.8
G3	19.08 ± 0.45	47.87 ± 1.17	62.47 ± 1.55	24.92 ± 0.47	39.87 ± 0.29	6.72 ± 0.2	24.02 ± 3.47	25.28 ± 4.75
G4	18.83 ± 0.89	47.58 ± 2.21	58.48 ± 2.06	23.15 ± 0.79	39.58 ± 0.72	7.2 ± 0.59	22.4 ± 3.08	22.88 ± 3.39
G1‐R	18.47 ± 0.42	44.75 ± 1.68	59.8 ± 0.84	24.72 ± 0.76	41.32 ± 1.56	6.88 ± 0.29	24.43 ± 1.3	26.84 ± 2.03
G4‐R	18.65 ± 0.55	45.88 ± 0.77	60.08 ± 1.6	24.45 ± 0.95	40.67 ± 0.84	7.08 ± 0.37	21.90 ± 2.13	25.92 ± 1.6

**Female rats (♀)**	**Hb (g/dL)**	**HCT (%)**	**MCV (fL)**	**MCH (pg)**	**MCHC (g/dL)**	**MPV (fL)**	**PT (sec)**	**APTT (sec)**

G1	18.48 ± 0.77	45.25 ± 1.77	59.07 ± 1.74	24.1 ± 0.97	40.77 ± 0.7	6.4 ± 0.25	24.02 ± 3.61	25.79 ± 2.68
G2	19.22 ± 0.56	47.28 ± 1.53	60.07 ± 1.15	24.4 ± 0.4	40.62 ± 0.31	6.42 ± 0.24	22.4 ± 4	26.65 ± 1.02
G3	18.98 ± 0.97	46.27 ± 2.35	58.8 ± 1.14	24.15 ± 0.7	41.02 ± 0.47	6.62 ± 0.43	23.35 ± 3.68	24.21 ± 6.03
G4	19.53 ± 0.98	48.15 ± 2.37	58 ± 0.7	23.53 ± 0.3	40.57 ± 0.31	6.45 ± 0.18	25.57 ± 3.06	25.57 ± 1.8
G1‐R	18.97 ± 0.62	44.62 ± 1.22	58.85 ± 1.2	25.02 ± 0.83	42.5 ± 1.26	6.42 ± 0.27	24.94 ± 2.99	26.52 ± 1.1
G4‐R	18.75 ± 0.38	45 ± 0.82	59.13 ± 1.18	24.65 ± 0.79	41.65 ± 0.61	6.62 ± 0.18	24.46 ± 1.38	27.63 ± 2.14

*Note:* Male and female Sprague‐Dawley rats were administered with Immunogrit doses (G1 (0 mg/kg body weight/day), G2 (100 mg/kg body weight/day), G3 (300 mg/kg body weight/day), and G4 (1000 mg/kg body weight/day) for 28 days and kept for 14‐day recovery period (G1‐R (0 mg/kg body weight/day) and G4‐R (1000 mg/kg body weight/day). The animals were analyzed for hematological parameters, such as hemoglobin content (Hb [g/dL]), hematocrit percentage (HCT [%]), mean corpuscular volume (MCV [fL]), mean corpuscular hemoglobin (MCH [pg]) content, mean corpuscular hemoglobin concentration (MCHC [g/dL]), mean platelet volume (MPV [fL]), prothrombin time (PT [sec]), and activated partial thromboplastin (APTT [sec]). Results are presented as mean ± standard deviation for all animal groups. Statistical analysis was performed using a one‐way ANOVA followed by Dunnett’s multiple comparisons post hoc test. In recovery groups, a *t* test was applied to all homogeneous data, whereas heterogeneous data were analyzed using Mann–Whitney’s test. A *p* value of < 0.05 was considered statistically significant.

Measurement of the blood serum biochemical parameters indicated the average presence of total bilirubin (male: 0.02 ± 0.02 mg/dL; female: 0.01 ± 0.02 mg/dL), cholesterol (male: 44.18 ± 4.50 mg/dL; female: 59.42 ± 5.03 mg/dL), calcium (male: 10.93 ± 0.37 mg/dL; female: 10.66 ± 0.11 mg/dL), urea (male: 28.11 ± 1.62 mg/dL; female: 37.55 ± 2.06 mg/dL), aspartate aminotransferase (male: 70.77 ± 4.47 U/L; female: 67.56 ± 8.82 U/L), alkaline phosphatase (male: 145.19 ± 17.30 U/L; female: 88.41 ± 11.81 U/L), triglycerides (male: 48.82 ± 3.27 mg/dL; female: 35.79 ± 8.99 mg/dL), glucose (male: 117.74 ± 7.97 mg/dL, female: 121.70 ± 6.74 mg/dL), phosphate (male: 7.76 ± 0.41 mg/dL, female: 6.38 ± 0.43 mg/dL), alanine aminotransferase (male: 25.92 ± 5.45 U/L, female: 22.55 ± 3.01 U/L), creatinine (male: 0.33 ± 0.02 mg/dL, female: 0.40 ± 0.05 mg/dL), albumin (male: 4.73 ± 0.28 g/dL, female: 5.11 ± 0.15 g/dL), total protein (Male: 6.57 ± 0.29 g/dL, female: 6.66 ± 0.22 g/dL), blood urea nitrogen (male: 13.13 ± 0.76 mg/dL, female: 17.55 ± 0.96 mg/dL), globulin (Male: 1.84 ± 0.11 g/dL, female: 1.55 ± 0.14 g/dL), sodium (male: 140.21 ± 0.70 mmol/L, female: 139.86 ± 1.38 mmol/L), potassium (male: 3.90 ± 0.11 mmol/L, female: 3.57 ± 0.20 mmol/L), chloride (male: 104.13 ± 3.23 mmol/L, female: 103.93 ± 1.97 mmol/L), and direct bilirubin (male: 0.04 ± 0.01 mg/dL, female: 0.05 ± 0.03 mg/dL) in all treatment and recovery animal groups (Table 5). The results indicated unaltered blood serum biochemical composition in both male and female SD rats following Immunogrit exposure.

**TABLE 5:** Blood serum biochemical analysis in Immunogrit‐exposed male and female rats.

Groups		T. Bil (mg/dL)	CHOL (mg/dL)	Ca (mg/dL)	Urea (mg/dL)	AST (U/L)	ALP (U/L)	TGL (mg/dL)	Glu (mg/dL)	Phos (mg/dL)	ALT (U/L)	Creat (mg/dL)	Alb (g/dL)	T. Pro (g/dL)	BUN (mg/dL)	Glob (g/dL)	Na (mmol/L)	K (mmol/L)	Cl (mmol/L)	D. Bil (mg/dL)
Male rats (♂)	G1	0.00 ± 0.01	38.43 ± 8.30	10.77 ± 0.28	26.99 ± 6.99	72.73 ± 5.76	151.97 ± 28.29	45.63 ± 9.99	109.00 ± 11.20	7.72 ± 0.49	24.73 ± 3.36	0.34 ± 0.04	4.91 ± 0.29	6.81 ± 0.34	12.61 ± 3.27	1.90 ± 0.12	139.13 ± 0.85	3.91 ± 0.19	105.68 ± 0.93	0.04 ± 0.02
	G2	0.00 ± 0.00	39.36 ± 5.13	10.68 ± 0.24	28.87 ± 6.52	78.27 ± 10.27	154.88 ± 44.65	47.55 ± 11.11	114.28 ± 29.04	7.46 ± 0.38	26.84 ± 5.23	0.33 ± 0.03	4.82 ± 0.18	6.80 ± 0.18	13.49 ± 3.05	1.98 ± 0.14	140.00 ± 0.59	3.75 ± 0.19	106.52 ± 0.44	0.04 ± 0.02
	G3	0.01 ± 0.02	46.02 ± 9.05	10.91 ± 0.74	26.61 ± 4.73	71.00 ± 6.35	149.86 ± 40.98	47.11 ± 8.41	110.70 ± 10.21	8.12 ± 0.69	29.99 ± 4.40	0.28 ± 0.08	4.87 ± 0.21	6.55 ± 0.24	12.43 ± 2.21	1.69 ± 0.32	139.93 ± 0.94	3.81 ± 0.22	106.15 ± 1.44	0.03 ± 0.02
	G4	0.01 ± 0.02	44.98 ± 8.07	11.59 ± 0.38^↑^	29.57 ± 3.97	65.20 ± 3.34	165.56 ± 18.76	54.16 ± 11.74	123.27 ± 23.65	8.38 ± 0.69	33.75 ± 5.18	0.33 ± 0.10	5.02 ± 0.11	6.82 ± 0.18	13.82 ± 1.86	1.80 ± 0.11	140.32 ± 0.53	3.94 ± 0.34	106.47 ± 1.42	0.05 ± 0.01
	G1‐R	0.03 ± 0.05	50.32 ± 8.97	10.55 ± 0.32	30.16 ± 3.34	68.98 ± 5.94	119.09 ± 12.07	51.45 ± 14.14	119.35 ± 7.99	7.39 ± 0.40	19.43 ± 3.05	0.33 ± 0.06	4.35 ± 0.16	6.25 ± 0.17	14.09 ± 1.56	1.90 ± 0.23	141.15 ± 1.07	4.08 ± 0.33	99.97 ± 1.16	0.04 ± 0.02
	G4‐R	0.04 ± 0.05	45.99 ± 6.93	11.09 ± 0.73	26.45 ± 5.01	68.43 ± 1.67	129.77 ± 24.4	47.01 ± 9.36	129.86 ± 20.03	7.48 ± 0.25	20.77 ± 4.24	0.34 ± 0.04	4.43 ± 0.21	6.19 ± 0.41	12.36 ± 2.34	1.76 ± 0.2	140.7 ± 1.44	3.92 ± 0.11	99.98 ± 0.44	0.06 ± 0.04

Female rats (♀)	G1	0.00 ± 0.01	63.83 ± 4.65	10.52 ± 0.26	37.26 ± 6.06	72.75 ± 11.94	94.95 ± 13.32	26.98 ± 5.50	131.09 ± 20.99	6.17 ± 0.60	21.58 ± 4.33	0.40 ± 0.03	5.14 ± 0.19	6.57 ± 0.28	17.41 ± 2.83	1.43 ± 0.10	139.18 ± 1.00	3.67 ± 0.42	105.93 ± 0.86	0.03 ± 0.02
	G2	0.00 ± 0.00	56.23 ± 5.17	10.64 ± 0.24	40.67 ± 8.79	74.73 ± 9.44	97.39 ± 18.10	31.43 ± 9.89	127.96 ± 16.84	6.57 ± 1.09	23.15 ± 1.51	0.48 ± 0.06 ↑	5.31 ± 0.25	6.75 ± 0.32	19.00 ± 4.11	1.44 ± 0.15	139.78 ± 1.25	3.76 ± 0.61	105.07 ± 1.75	0.03 ± 0.02
	G3	0.01 ± 0.02	60.77 ± 4.15	10.72 ± 0.30	35.37 ± 5.88	77.20 ± 15.57	93.92 ± 9.45	33.80 ± 6.92	117.31 ± 11.60	6.84 ± 0.61	22.25 ± 3.90	0.39 ± 0.03	5.15 ± 0.14	6.92 ± 0.13	16.53 ± 2.75	1.77 ± 0.16 ↑	138.75 ± 0.29	3.52 ± 0.30	104.95 ± 0.54	0.04 ± 0.02
	G4	0.00 ± 0.00	51.04 ± 12.02	10.86 ± 0.30	35.26 ± 2.38	66.22 ± 2.76	94.70 ± 13.46	31.62 ± 4.73	122.71 ± 7.11	6.82 ± 0.35	28.13 ± 4.67^↑^	0.39 ± 0.05	5.19 ± 0.12	6.88 ± 0.17	16.48 ± 1.11 ↑	1.68 ± 0.18 ↑	138.40 ± 0.91	3.77 ± 0.30	104.63 ± 1.03	0.05 ± 0.02
	G1‐R	0.02 ± 0.03	64.32 ± 7.76	10.63 ± 0.14	38.53 ± 8.41	56.12 ± 2.12	66.65 ± 8.71	52.51 ± 9.81	117.01 ± 6.22	5.99 ± 0.38	19.43 ± 3.05	0.34 ± 0.08	4.91 ± 0.07	6.42 ± 0.13	18.00 ± 3.93	1.52 ± 0.16	141.27 ± 0.76	3.25 ± 0.12	100.95 ± 0.81	0.09 ± 0.02
	G4‐R	0.04 ± 0.05	60.35 ± 15.86	10.57 ± 0.26	38.20 ± 6.90	58.32 ± 6.59	82.85 ± 10.20 ↑	38.37 ± 9.47 ↓	114.11 ± 9.11	5.86 ± 0.50	20.77 ± 4.24	0.41 ± 0.06 ↑	4.97 ± 0.25	6.44 ± 0.35	17.85 ± 3.22	1.47 ± 0.15	141.8 ± 1.3	3.44 ± 0.21	102.02 ± 1.11	0.08 ± 0.02

*Note:* Male and female Sprague‐Dawley rats in G1 (0 mg/kg body weight/day), G2 (100 mg/kg body weight/day), G3 (300 mg/kg body weight/day), and G4 (1000 mg/kg body weight/day) groups were administered with 28‐day Immunogrit doses and kept for the 14‐day recovery period (G1‐R (0 mg/kg body weight/day) and G4‐R (1000 mg/kg body weight/day)). All animals were analyzed for total bilirubin (T. Bil [mg/dL]), cholesterol (CHOL [mg/dL]), calcium (Ca [mg/dL]), urea [mg/dL], aspartate aminotransferase (AST [U/L]), alkaline phosphatase (ALP [U/L]), triglycerides (TGL [mg/dL]), glucose (Glu [mg/dL]), phosphate (Phos [mg/dL]), alanine aminotransferase (ALT [U/L]), creatinine (Creat [mg/dL]), albumin (Alb [g/dL]), total protein (T. Pro [g/dL]), blood urea nitrogen (BUN [mg/dL]), globulin (Glob [g/dL]), sodium (Na [mmol/L]), potassium (K [mmol/L]), chloride (Cl [mmol/L]), and direct bilirubin (D. Bil [mg/dL]) in whole blood serum. Results are presented as mean ± standard deviation for all animal groups. Statistical analysis was performed using a one‐way ANOVA followed by Dunnett’s multiple comparisons post hoc test. In recovery groups, a *t* test was applied to all homogeneous data, whereas heterogeneous data were analyzed using Mann–Whitney’s test. A *p* value of < 0.05 was considered statistically significant.

Urine biochemical analysis indicated the absence of any renal damage in the Immunogrit‐treated and recovery animal groups. Biochemical parameters in Immunogrit‐exposed male and female rats showed average urine volume (male 10.31 ± 0.08 mL; female 10.04 ± 0.17 mL), bilirubin (both male and female 0.00 ± 0.00 mg/dL), ketone (male 16.25 ± 6.10 mg/dL; female 0.21 ± 0.42 mg/dL), specific gravity (both male and female 1.02 ± 0.00), RBC counts (male 0.84 ± 0.96 RBCs/μL; female 0.00 ± 0.00 RBCs/μL), urine pH condition (male 7.58 ± 0.38; female 7.40 ± 0.24), protein levels (male 17.50 ± 6.16 mg/dL; female 1.25 ± 1.59 mg/dL), urobilinogen level (both male and female 0.10 ± 0.00 mg/dL), glucose level (both male and female 0.00 ± 0.00 mg/dL), leukocyte count (both male and female 0.00 ± 0.00 WBCs/μL), and nitrite level (male 0.00 ± 0.00 mg/dL; female 0.01 ± 0.01 mg/dL), indicating that they were within the historical reference range reported for the study center (Table 6). Male SD rats had a higher urinary excretion of total protein than female rats [30]. Variable presence of urinary proteins has been reported in normal male SD rats ranging 191 ± 169 mg/dL [31]. Hence, the variable urinary protein levels observed in the male SD rats in this study may be incidental in nature and independent of Immunogrit treatment conditions. G1‐R and G4‐R male and female animals also showed no change in their urine biochemical parameters, indicating the absence of any latent development of renal toxicity following exposure to Immunogrit (Table 6).

**TABLE 6:** Urine biochemical analysis in Immunogrit‐exposed male and female Sprague‐Dawley rats.

Groups		Volume (mL)	Bilirubin (mg/dL)	Ketone (mg/dL)	Specific gravity	RBCs (μL)	pH	Protein (mg/dL)	Urobilinogen (mg/dL)	Glucose (mg/dL)	WBCs (μL)	Nitrite (mg/dL)
Male rats (♂)	G1	10.25 ± 0.52	0.00 ± 0.00	22.50 ± 21.39	1.02 ± 0.00	1.67 ± 4.08	7.67 ± 0.41	15.00 ± 12.25	0.10 ± 0.00	0.00 ± 0.00	0.00 ± 0.00	0.00 ± 0.00
	G2	10.33 ± 0.41	0.00 ± 0.00	13.33 ± 18.35	1.02 ± 0.01	1.67 ± 4.08	8.00 ± 0.63	23.33 ± 10.33	0.10 ± 0.00	0.00 ± 0.00	0.00 ± 0.00	0.00 ± 0.00
	G3	10.42 ± 0.38	0.00 ± 0.00	9.17 ± 2.04	1.02 ± 0.01	0.00 ± 0.00	7.08 ± 0.38	21.67 ± 13.29	0.10 ± 0.00	0.00 ± 0.00	0.00 ± 0.00	0.00 ± 0.00
	G4	10.25 ± 0.27	0.00 ± 0.00	20.00 ± 23.45	1.02 ± 0.00	0.00 ± 0.00	7.58 ± 0.66	10.00 ± 10.95	0.10 ± 0.00	0.00 ± 0.00	0.00 ± 0.00	0.00 ± 0.00
	G1R	9.83 ± 0.52	0.00 ± 0.00	6.67 ± 5.16	1.01 ± 0.00	0.00 ± 0.00	8.40 ± 0.22	13.33 ± 8.16	0.10 ± 0.00	0.00 ± 0.00	5.00 ± 5.48	0.00 ± 0.00
	G4R	9.83 ± 0.52	0.00 ± 0.00	30.00 ± 21.91	1.01 ± 0.00	0.00 ± 0.00	8.33 ± 0.29	41.67 ± 28.58	0.10 ± 0.00	0.00 ± 0.00	5.00 ± 5.48	0.00 ± 0.00

Female rats (♀)	G1	10.08 ± 0.58	0.00 ± 0.00	0.00 ± 0.00	1.02 ± 0.01	0.00 ± 0.00	7.08 ± 0.92	0.00 ± 0.00	0.10 ± 0.00	0.00 ± 0.00	0.00 ± 0.00	0.00 ± 0.00
	G2	10.00 ± 0.45	0.00 ± 0.00	0.83 ± 2.04	1.02 ± 0.00	0.00 ± 0.00	7.67 ± 0.26	3.33 ± 5.16	0.10 ± 0.00	0.00 ± 0.00	0.00 ± 0.00	0.02 ± 0.04
	G3	9.83 ± 0.41	0.00 ± 0.00	0.00 ± 0.00	1.02 ± 0.01	0.00 ± 0.00	7.42 ± 0.74	1.67 ± 4.08	0.10 ± 0.00	0.00 ± 0.00	0.00 ± 0.00	0.00 ± 0.00
	G4	10.25 ± 0.27	0.00 ± 0.00	0.00 ± 0.00	1.02 ± 0.00	0.00 ± 0.80	7.42 ± 0.00	0.00 ± 0.00	0.10 ± 0.00	0.00 ± 0.00	0.00 ± 0.00	0.00 ± 0.00
	G1R	9.75 ± 0.52	0.00 ± 0.00	0.00 ± 0.00	1.02 ± 0.01	0.00 ± 0.00	8.08 ± 0.66	0.00 ± 0.00	0.10 ± 0.00	0.00 ± 0.00	0.00 ± 0.00	0.00 ± 0.00
	G4R	9.75 ± 0.52	0.00 ± 0.00	0.00 ± 0.00	1.01 ± 0.00	0.00 ± 0.00	8.25 ± 0.61	0.00 ± 0.00	0.10 ± 0.00	0.00 ± 0.00	0.00 ± 0.00	0.00 ± 0.00

*Note:* Sprague‐Dawley rats were treated with varying dosages of Immunogrit formulation (G1 (0 mg/kg body weight/day), G2 (100 mg/kg body weight/day), G3 (300 mg/kg body weight/day), and G4 (1000 mg/kg body weight/day)) daily for 28 days. The recovery group (G1‐R (0 mg/kg body weight/day) and G4‐R (1000 mg/kg body weight/day)) animals were kept under observation for 14 days. Urine collected in metabolic cages was analyzed for volume (mL), bilirubin content (Bil [mg/dL]), ketone content (mg/dL), specific gravity (SG), red blood corpuscles (RBCs/μL), pH condition, protein content (mg/dL), urobilinogen content (mg/dL), glucose content (mg/dL), white blood cells (WBCs/μL), and nitrite (mg/dL) kept under observation for latent toxicological responses. Treatment and recovery animal groups were placed in metabolic cages on Days 28 and 42, respectively, and urine was collected in thymol blue crystals containing beakers. Results are presented as mean ± standard deviation for all animal groups. Statistical analysis was performed using a one‐way ANOVA followed by Dunnett’s multiple comparisons post hoc test. In recovery groups, a *t* test was applied to all homogeneous data, whereas heterogeneous data were analyzed using Mann–Whitney’s test. A *p* value < 0.05 was considered statistically significant.

**FIGURE 5:**
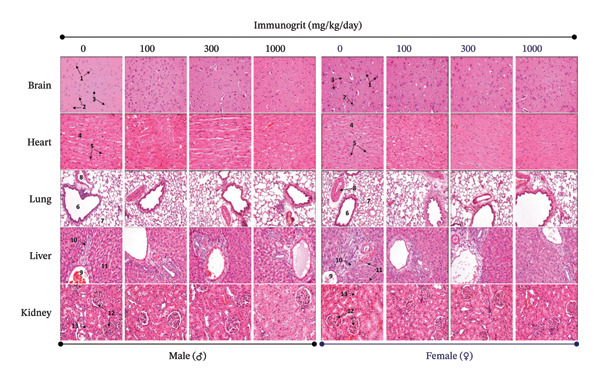
Histopathological analysis of the vital organs in Immunogrit‐exposed male and female Sprague‐Dawley rats. Representative histopathological images obtained from the five vital organs (brain [1—neurons; 2—microglia; 3—astrocytes], heart [4—striations; 5—central nucleus], lung [6—bronchioles, 7—alveolus, 8—blood vessel], liver [9—portal vein, 10—bile duct, 11—hepatocyte], and kidney [12—renal corpuscles, 13—thick conducting duct]) of the male and female Sprague‐Dawley rats treatment group: vehicle control (G1), 100 mg/kg body weight (G2), 300 mg/kg body weight (G3), 1000 mg/kg body weight (G4). No histopathological abnormalities were observed in all the treatment groups.

Both male and female animal groups exposed to Immunogrit showed no change in their terminal body and absolute organ weights during 28 treatment days and 14‐day recovery period, compared to their respective controls (G1, G1‐R) (Table 7). Histopathological analysis of the Immunogrit‐exposed male and female SD rat groups showed a regular brain architecture with neurons, microglia, and astrocytes (Figure 5). Heart tissue histopathology showed normal striations and central nuclear regions in all animal groups. Lung tissue section showed the presence of unaltered bronchioles, alveolus, and blood vessel in the Immunogrit‐exposed male and female animal groups (Figure 5). Liver tissue sections obtained from the Immunogrit‐exposed animals showed the presence of unmodified portal vein, bile duct, and hepatocytes (Figure 5). No Immunogrit exposure–associated abnormalities were observed in kidney tissue sections obtained from different animal groups showing the presence of renal corpuscles and thick conducting duct (Figure 5). No histopathological abnormalities were identified in secondary organs obtained from Immunogrit‐treated male and female animal groups (data not shown).

**TABLE 7:** Terminal body and absolute organ weights in Immunogrit‐exposed male and female Sprague‐Dawley rats.

Group		Terminal body weight (gm)	Adrenals (gm)	Thymus (gm)	Spleen (gm)	Epididymides (gm)	Testis (gm)	Brain (gm)	Heart (gm)	Kidneys (gm)	Liver (gm)	Prostrate (gm)	Seminal vesicle (gm)
Male rats (♂)	G1	361.15 ± 15.74	0.07 ± 0.01	0.60 ± 0.12	0.85 ± 0.10	0.97 ± 0.07	3.42 ± 0.23	2.06 ± 0.03	1.46 ± 0.13	2.91 ± 0.17	13.11 ± 1.44	0.94 ± 0.15	1.27 ± 0.15
	G2	353.44 ± 24.44	0.08 ± 0.02	0.54 ± 0.10	0.77 ± 0.12	0.98 ± 0.10	3.21 ± 0.27	2.12 ± 0.05	1.46 ± 0.13	2.89 ± 0.35	13.31 ± 1.82	0.95 ± 0.05	1.19 ± 0.04
	G3	357.85 ± 26.70	0.07 ± 0.02	0.62 ± 0.18	0.78 ± 0.13	0.99 ± 0.03	3.26 ± 0.21	2.06 ± 0.12	1.49 ± 0.16	2.97 ± 0.26	13.35 ± 2.13	0.97 ± 0.17	1.30 ± 0.14
	G4	343.56 ± 28.05	0.07 ± 0.01	0.54 ± 0.16	0.84 ± 0.21	0.99 ± 0.05	3.26 ± 0.12	2.04 ± 0.09	1.39 ± 0.15	2.78 ± 0.22	12.33 ± 1.82	0.93 ± 0.08	1.09 ± 0.13
	G1R	399.55 ± 17.31	0.06 ± 0.01	0.52 ± 0.10	0.80 ± 0.15	1.30 ± 0.08	3.28 ± 0.14	2.16 ± 0.14	1.59 ± 0.17	3.05 ± 0.32	13.84 ± 1.27	1.17 ± 0.12	1.29 ± 0.31
	G4R	411.44 ± 45.14	0.06 ± 0.01	0.58 ± 0.16	0.89 ± 0.09	1.26 ± 0.09	3.44 ± 0.20	2.10 ± 0.09	1.44 ± 0.14	3.05 ± 0.17	13.10 ± 1.53	1.0093 ± 0.10 ↓	1.26 ± 0.04

**Group**			**Terminal body weight (gm)**	**Adrenals (gm)**	**Thymus (gm)**	**Spleen (gm)**	**Uterus/cervix (gm)**	**Ovaries (gm)**		**Brain (gm)**	**Heart (gm)**	**Kidneys (gm)**	**Liver (gm)**

Female rats (♀)		G1	235.61 ± 21.62	0.07 ± 0.01	0.59 ± 0.18	0.54 ± 0.07	0.58 ± 0.24	0.17 ± 0.02		2.02 ± 0.10	1.03 ± 0.12	2.05 ± 0.15	8.82 ± 0.98
		G2	228.37 ± 19.61	0.07 ± 0.01	0.52 ± 0.11	0.54 ± 0.09	0.69 ± 0.19	0.14 ± 0.02		2.02 ± 0.06	0.97 ± 0.07	1.92 ± 0.13	8.57 ± 1.44
		G3	227.60 ± 17.11	0.08 ± 0.01	0.51 ± 0.09	0.62 ± 0.05	0.54 ± 0.21	0.15 ± 0.03		2.00 ± 0.09	1.02 ± 0.09	2.03 ± 0.21	8.68 ± 0.98
		G4	216.56 ± 14.14	0.07 ± 0.01	0.43 ± 0.09	0.60 ± 0.09	0.57 ± 0.22	0.1217 ± 0.02 ↓		2.00 ± 0.06	0.88 ± 0.09	1.92 ± 0.11	8.24 ± 0.72
		G1R	259.33 ± 23.02	0.07 ± 0.01	0.46 ± 0.07	0.55 ± 0.10	0.57 ± 0.18	0.14 ± 0.02		2.11 ± 0.07	1.05 ± 0.12	2.05 ± 0.18	9.07 ± 1.41
		G4R	238.30 ± 19.25	0.07 ± 0.01	0.50 ± 0.08	0.57 ± 0.07	0.50 ± 0.06	0.14 ± 0.02		2.06 ± 0.09	0.98 ± 0.08	2.10 ± 0.20	8.89 ± 0.89

*Note:* Male and female Sprague‐Dawley rats (G1 (0 mg/kg body weight/day), G2 (100 mg/kg body weight/day), G3 (300 mg/kg body weight/day), and G4 (1000 mg/kg body weight/day)) were treated with varying dosages of Immunogrit medicine daily for 28 days. The recovery group animals (G1‐R (0 mg/kg body weight/day) and G4‐R (1000 mg/kg body weight/day)) were further kept under observation for 14 days to observed any induction of latent toxicological responses. On the terminal day, animals were euthanized, and organs were harvested and weighed. Results are presented as mean ± standard deviation for all animal groups. Statistical analysis was performed using a one‐way ANOVA followed by Dunnett’s multiple comparisons post hoc test. In recovery groups, a *t* test was applied to all homogeneous data, whereas heterogeneous data were analyzed using Mann–Whitney’s test. A *p* value of < 0.05 was considered statistically significant.

Mutagenicity analysis of Immunogrit and its S9 metabolites did not induce any mutagenic effect in *S. typhimurium* TA 1535, TA 1537, TA 98, TA 100, and *E. coli* WP2uvrA strains up to the highest tested concentration of 5 mg/plate (Table 8). The assay was validated using standard mutagens (Table 8). The MR for Immunogrit and its S9 metabolites was below 2.0, indicating their nonmutagenic potential (Table 8).

**TABLE 8:** Ames bacterial mutagenicity test of Immunogrit and its S9 fraction metabolites.

Immunogrit dose (mg/plate)		*Salmonella typhimurium*								*Escherichia coli* WP2uvrA	
		TA 1535		TA 1537		TA 98		TA 100			
		Mean ± SD	# MR	Mean ± SD	# MR	Mean ± SD	# MR	Mean ± SD	# MR	Mean ± SD	# MR
Without S9 fraction	DMSO	9.3 ± 0.6	1.0	7.3 ± 1.5	1.0	11.3 ± 0.6	1.0	63.7 ± 5.1	1.0	16.3 ± 1.5	1.0
	0.05	6.7 ± 0.6	0.9	8.0 ± 3.5	1.1	7.0 ± 2.0	0.6	66.3 ± 6.8	1.0	15.3 ± 0.6	0.9
	0.15	6.7 ± 1.5	0.9	8.3 ± 2.5	1.1	9.7 ± 1.5	0.9	67.7 ± 3.8	1.1	15.0 ± 5.3	0.9
	0.5	8.0 ± 2.0	1.1	4.0 ± 1.7	0.5	6.7 ± 2.5	0.6	61.0 ± 2.6	1.0	13.3 ± 3.8	0.8
	1.5	8.3 ± 2.5	1.1	8.0 ± 1.7	1.1	8.0 ± 1.0	0.7	67.0 ± 4.0	1.1	12.7 ± 3.8	0.8
	5	7.7 ± 1.5	1.0	7.7 ± 0.6	1.0	5.0 ± 2.6	0.4	65.3 ± 2.5	1.0	18.0 ± 3.6	1.1
	Standard mutagen^Ψ^	167.7 ± 6.1	22.9^∗^	139.3 ± 18.0	19.0^∗^	95.7 ± 14.6	8.4^∗^	257.3 ± 21.0	4.0^∗^	334.3 ± 24.8	20.5^∗^

With S9 fraction	DMSO	9.0 ± 1.0	1.0	22.0 ± 3.0	1.0	38.3 ± 2.1	1.0	68.0 ± 2.0	1.0	50.0 ± 5.0	1.0
	0.05	10.3 ± 1.5	1.1	11.0 ± 8.9	0.5	42.7 ± 4.2	1.1	76.0 ± 4.4	1.1	43.0 ± 2.6	0.9
	0.15	8.3 ± 3.1	0.9	18.3 ± 10.0	0.8	40.6 ± 5.1	1.1	71.0 ± 9.5	1.0	39.3 ± 8.1	0.8
	0.5	11.3 ± 8.1	1.3	24.0 ± 1.0	1.1	37.7 ± 2.5	1.0	68.0 ± 3.0	1.0	41.3 ± 4.9	0.8
	1.5	8.7 ± 2.1	1.0	17.3 ± 3.8	0.8	41.0 ± 1.0	1.1	72.7 ± 6.7	1.1	36.0 ± 2.0	0.7
	5	10.0 ± 1.0	1.1	20.3 ± 8.6	0.9	42.0 ± 4.0	1.1	69.0 ± 5.2	1.0	40.3 ± 5.5	0.8
	Standard mutagen (Ψ)	67.0 ± 3.0	7.4^∗^	139.7 ± 11.0	6.3^∗^	598.3 ± 63.0	15.6^∗^	671.6 ± 46.5	9.9^∗^	273.0 ± 22.6	5.5^∗^

*Note:* Bacterial mutagenicity analysis showing the average number of revertants per plate inoculated with *Salmonella typhimurium* strains TA 1535, TA 1537, TA 98 and TA 100 and *Escherichia coli* WP2uvrA strains following treatment with Immunogrit in the presence or absence of S9 fraction. Standard mutagen (Ψ) without S9 fraction treatments: 4‐nitroquinoline (0.15 μg/plate) in TA 98; sodium azide (0.5 μg/plate) in TA 100 and TA 1535, 9‐aminoacridine (50 μg/plate) in TA 1537, and 4‐nitroquinoline (0.5 μg/plate) in *E. coli* WP2uvrA. 2‐Aminoanthracene (20 μg/plate) was used as the standard mutagen in S9 fraction metabolic model for all bacterial strains. Mutagenicity ratio (MR) = number of revertant colonies in Immunogrit or mutagen/number of revertant colonies in vehicle (DMSO). #Mean of revertant colonies per plate (*n* = 3).

^∗^indicates that the value is significant (*p* value < 0.05) compared to vehicle (DMSO).

Overall, the results indicated that the 28‐day repeated subacute exposure of the male and female SD rats to Immunogrit did not induce any immediate or latent physiological, behavioral, neurological, or histopathological abnormalities. Based on the study outcome, the “NOAEL” for Immunogrit was found at 1000 mg/kg body weight/day. Ames assay‐based mutagenicity analysis did not show any mutagenic effects from Immunogrit and its S9 fraction metabolites up to the highest tested concentration of 5 mg/plate.

## 4. Discussion

Increasing global dependencies of patients on herbal medicines make it important to assess their safety using standardized toxicological methods [4]. In the present study, subacute toxicological profiling of the immunomodulatory Ayurvedic polyherbal medicine “Immunogrit” was done in male and female SD rats following repeated 28‐day oral exposure. The study outcomes indicated that oral exposure to Immunogrit did not introduce any immediate or latent adverse effects in male or female SD rats up to the highest tested dose of 1000 mg/kg body weight/day. The results established 1000 mg/kg body weight/day as the “NOAEL” dose for Immunogrit.

The UPLC/MS‐QToF–based analysis showed the presence of a diverse range of phytochemicals belonging to phenolic acids, flavonoids, proanthocyanidins, lignans, saponins, and triterpenoids groups in Immunogrit. Several of these phytochemicals have been screened individually for their toxicological profile. Variya et al. observed the lethal dose 50% (LD50) of gallic acid to be > 2000 mg/kg in male and female albino mice [32]. Tasaki et al. observed the NOAEL for ellagic acid in male F344 rats at 3011 mg/kg body weight/day and 778 mg/kg body weight/day in female rats [33]. Epigallocatechin gallate was found to have NOAEL up to 500 mg/kg body weight/day in rats over a period of 13 days [34]. However, it induced mortality, morbidity, and skin and eye irritation at higher doses in different animal models [34]. Toxicity analysis of scopoletin has shown it to be safe up to 2000 mg/kg [35].

Earlier studies have reported plant components of Immunogrit, such as *P. tuberosa* (Roxb. ex Willd.) DC., *A. racemosus* Willd., *S. cordifolia* L., *M. pruriens* (L.) DC., and *W. somnifera* (L.) Dunal to be safe, when tested independently [12–16]. However, concerns have been raised regarding the hepatotoxic potential of *D. bulbifera* L., attributed to its metabolic by‐products from cytochrome P450‐metabolism and *R. procera* Wall. inducing toxicity at doses of 5000 mg/kg body weight [17, 18, 36, 37]. However, in our study, the absence of any observable alterations in the liver enzyme (ALT, AST, and ALP) levels in the blood serum of the Immunogrit‐exposed animals indicated a healthy hepatic functioning. Urinanalysis confirmed the renal safety of Immunogrit in male and female SD rats with normal pH, specific gravity, and the absence of proteinuria, hematuria, or glycosuria. Similarly, stable creatinine, urea, and electrolyte levels suggested normal kidney functioning in these Immunogrit‐treated animals. The histopathological profiling further confirmed the hepatic and renal system safety of Immunogrit medicine within the tested dose range. Overall, the level of hepatic and renal biomarkers observed in the normal and Immunogrit‐treated animals concurred with the ranges reported by earlier studies in male and female SD rats [27–29].

Throughout the 28‐day Immunogrit treatment and subsequent 14‐day recovery period, no mortality, morbidity, or abnormal clinical signs were observed in both the male and female SD rats. Also, no change was observed in the animals’ body weight, food consumption, and general activity patterns, indicating that the consumed polyherbal medicine was safe and did not affect the SD rats’ normal physiological and metabolic behavior. An ophthalmological examination of the Immunogrit‐treated animals indicated no ocular/neuronal damage induction. Additionally, the absence of any functional and neurological adverse effects in terms of posture, reactivity, and exploratory behavior, with no evidence of ataxia, or convulsions throughout the study period provided evidence for the biosafety of Immunogrit medicine up to the tested concentration of 1000 mg/kg body weight/day. Hence, the results confirmed the absence of any Immunogrit‐induced adverse impact on the animal’s motor coordination, reflex responses, and autonomic functions.

Hematological analysis in the Immunogrit‐treated male and female SD rats and the recovery animal groups indicated the absence of any abnormality in differential leukocyte counts, blood hemoglobin content, platelet function, or coagulation parameters. Stability of blood coagulation markers, including prothrombin time and activated partial thromboplastin time, further confirms the low‐risk levels of Immunogrit in the induction of hemorrhages. Hence, the results indicated that Immunogrit lacked any immunomodulatory impact. No Immunogrit treatment‐related adverse histopathological impacts were observed in the vital and secondary organs. Tissue architecture remained intact across all treatment groups, with no evidence of inflammation, necrosis, fibrosis, or cellular degeneration.

However, a majority of Immunogrit plant components have not been tested for their mutagenic properties. *D. bulbifera* L. extract induces liver toxicity through cytochrome P450 3A–based metabolism of *cis*‐enedial intermediates [17]. *A. racemosus* Willd. and *C. borivilianum* Santapau & R.R. Fern. were found to be nonmutagenic using the bacterial Ames test [38, 39]. In fact, the treatment of *C. borivilianum* Santapau & R.R. Fern. has been reported to reduce the natural formation of chromosomal aberration through antioxidant activities [38]. In this study, Immunogrit and its S9 metabolites showed non‐mutagenic potential in the four *S. typhimurium* strains and one *E. coli* WP2uvrA strain.

The study established NOAEL for Immunogrit at 1000 mg/kg body weight/day, indicating that the long‐termconsumption of Immunogrit would be safe. Considering species‐specific differences in metabolism, still appropriate dose–response extrapolation must be done through clinical trials. Furthermore, this study confirms the safety of Immunogrit medicine at a subacute dosage, further investigations are warranted to determine its chronic toxicity, reproductive toxicity, and potential interactions with other medications.

## 5. Conclusion

In summary, our findings demonstrate that Immunogrit was well‐tolerated in the male and female SD rats and did not induce mortality, behavioral abnormalities, hematological disruptions, or organ toxicity at doses up to 1000 mg/kg body weight/day over 28 days. Immunogrit also did not induce any latent effects during the recovery period. In addition, Immunogrit was found to be nonmutagenic in the Ames assay. Finally, the NOAEL for Immunogrit was determined at 1000 mg/kg body weight/day and provides a basis for its further nonclinical and clinical investigation.

## Author Contributions

Acharya Balkrishna: conceptualization, planning, supervision, resources, and writing–review and editing; Kunal Bhattacharya: data curation, formal analysis, writing original draft, and visualization; Sudeep Verma: methodology, investigation, data curation, and formal analysis; Himanshu Jangid: methodology, investigation, data curation, and formal analysis; Savita Lochab: methodology, investigation, data curation, and formal analysis; Anurag Varshney: project administration, conceptualization, visualization, supervision, and writing–review and editing.

## Funding

This study was funded internally by the Patanjali Research Foundation Trust, Haridwar, India.

## Conflicts of Interest

Acharya Balkrishna is a trustee in the Divya Yog Mandir Trust, Haridwar, India, that governs Divya Pharmacy, Haridwar, and holds an honorary managerial position at Patanjali Ayurved Ltd, Haridwar, India. These organizations were not involved in any aspects of this study. Kunal Bhattacharya, Sudeep Verma, Himanshu Jangid, Savita Lochab, and Anurag Varshney are employed at the Patanjali Research Foundation, which is governed by the Patanjali Research Foundation Trust (PRFT), Haridwar, Uttarakhand, India, a not‐for‐profit organization. In addition, Anurag Varshney is an Adjunct Professor in the Department of Allied and Applied Sciences, University of Patanjali, Haridwar, India. The other authors declare no conflicts of interest.

## Data Availability

The data supporting the findings of this study are available from the corresponding author upon reasonable request.
